# Parents’ Knowledge, Attitude, and Practice Regarding Febrile Convulsion in Children in Riyadh, Saudi Arabia

**DOI:** 10.7759/cureus.47314

**Published:** 2023-10-19

**Authors:** Abdulaziz Almousa, Dayel Alshahrani, Muath S Almubarak, Abdulrahman Alothman, Abdulrahman M Alrashoudi, Abdulaziz Alsharif, Ibrahim A Almehaidib, Abdulaziz I Almohanna

**Affiliations:** 1 College of Medicine, Imam Mohammad Ibn Saud Islamic University, Riyadh, SAU; 2 Research, King Fahad Medical City, Riyadh, SAU; 3 Pediatric Infectious Diseases, King Fahad Medical City, Riyadh, SAU; 4 Medical School, Imam Mohammad Ibn Saud Islamic University, Riyadh, SAU; 5 Medical School, ‏Imam Mohammad Ibn Saud Islamic University, Riyadh, SAU

**Keywords:** concern, practice, children, parents, attitude, knowledge, febrile convulsion

## Abstract

Background

Fever is a common presentation in pediatric age groups and is the most common reason for healthcare visits. Parents’ knowledge is essential for early presentation to healthcare facilities and to prevent possible complications. Studies suggest that febrile convulsions (FCs) often cause stress and anxiety for parents, who often assume it may cause brain damage, future epilepsy attacks, mental retardation, physical injury, and even death. This study aims to assess the knowledge, attitude, and practices of FCs among parents.

Methodology

This cross-sectional study was conducted among parents living in Riyadh, Saudi Arabia, with children younger than 14 years of age. A self-administered questionnaire was distributed among the selected population using an online survey. The questionnaire included socio-demographic data, family experiences of FC, practice toward a child with FC, and questions assessing knowledge, attitude, and concerns.

Results

Of the 415 recruited parents, 350 (84.3%) were mothers, and 56.4% were government employees. Regarding monthly income, 172 (41.4%) were earning more than 15,000 SAR per month. The prevalence of parents with having a child with FC was 33%. Overall, 369 (88.9%) had poor knowledge, 41 (9.9%) had moderate knowledge, and only five (1.2%) had good knowledge levels. Regarding attitude, more than one-third (200, 35.2%), had a negative attitude, 205 (49.4%) were neutral, and the rest had positive attitude levels (64, 15.4%). Increased knowledge and attitude toward FC were associated with having a child or siblings of a child with FC. Regarding the practice and the plan of action for future FC occurrence, the most common was to rush the child to a doctor (94, 68.6%), followed by lowering the child’s body temperature (78, 56.9%), and placing the child on his/her side (54, 39.4%).

Conclusions

There was an optimistic attitude toward a child with FC but knowledge was deficient. However, parents who had a child or siblings experiencing FC were more knowledgeable, while parents with higher education and better income tended to exhibit better attitudes when managing a child with FC. Further investigations are needed to establish the level of understanding and perspectives of parents when managing a child with FC.

## Introduction

Fever is a common presentation in pediatric age groups and is the most common reason for healthcare visits [1]. The American Academy of Pediatrics defines a febrile seizure as “a seizure accompanied by fever (temperature ≥100.4°F or 38°C by any method), without central nervous system infection, that occurs in infants and children six through 60 months of age” [2]. Parents’ knowledge about febrile seizures and their management is considered an essential part of the treatment [2]. In addition, many studies suggest that febrile convulsions (FCs) often cause stress and anxiety for parents, who often assume it may cause brain damage, future epilepsy attacks, mental retardation, physical injury, and even death [2,3]. FCs can occur in 2% to 4% of all children, with 90% occurring between six months to three years [3]. Although febrile seizure is usually benign, a 12-year follow-up study showed that children with FC have a 6% chance of developing epilepsy and a 10% chance of developing neurological problems [3].

Mothers can exhibit abnormal behaviors due to a lack of information about the disease and its high recurrence rate. Some mothers become confused and afraid when their children have a fever and lose control, preventing them from taking action to control the rage and its complications [4,5]. The best strategy to prevent this illness is properly controlling a child's fever, especially in babies. Trained mothers can avoid problems with easy prevention, fever control, foot washing (with cool water), and the correct use of anti-fever medications [6,7].

Internationally, In 2009 a study was performed through a questionnaire administered to mothers of children who came to health departments for a regular 18-month well-baby assessment [8]. The study assessed and compared mothers’ understanding and perceptions of fever, fever management practices, and the knowledge sources of mothers of children with and without a history of febrile seizures.

A total of 386 answers were analyzed. The knowledge of mothers of children with past febrile seizures was better, who noted that high fever caused febrile seizures and antipyretics would stop it rather than mothers of children without past experience. A smaller number of mothers of children with past febrile seizures than mothers with no experience believed that high fever induced brain damage and antipyretics would protect the child from getting worse and warm the child’s body during fever attacks. Multiple mothers in the two classes expressed that they assumed physicians as their primary knowledge reference. Partners and parents were called as information references among mothers of children with a history of febrile seizures. At the same time, books and the Internet were referred to in the other group.

Furthermore, mothers of children with past febrile seizures illustrated an increased accuracy rate in their understanding of fever than those without experience. Mothers of children with past febrile seizures used personal contact, whereas those in the other class depended on mass communication for health knowledge. Hence, delivering factual knowledge to families is essential to provide mothers with proper knowledge and strong support [8].

Locally, a cross-sectional study was performed in 2020 in the Al Qassim region. An Arabic online questionnaire study was conducted among parents to evaluate knowledge and attitude and to specify practices concerning children with FCs.

A total of 447 participants’ outcomes reflected critical results; 67.8% of parents had inadequate knowledge, where unfavorable attitudes were identified for 57%, and 58.4% had good practice.

Generally, parents from Al Qassim with children with FCs were found to have moderate practice and suboptimal knowledge and attitude. Their practices were found to be moderate. Educated fathers having more than three children remarkably impacted the parents’ knowledge of FC [9].

This cross-sectional study aims to objectively assess the knowledge, attitude, and practice of FCs among parents in Riyadh, Saudi Arabia, while also exploring the association with socioeconomic status. The study aims to identify areas where knowledge is lacking and develop future online educational activities to target those areas.

## Materials and methods

In this cross-sectional study, data were collected from voluntary participants from the general population using a validated questionnaire after they had provided informed consent and declared the authenticity of the information provided. Participants were recruited using a convenient sampling technique. With their permission, the data were gathered using an online questionnaire adapted from Huang et al. [10]. A translated copy into Arabic was validated. The questionnaire was constructed on Google Forms and distributed via digital platforms to parents with children under 14 years of age in Riyadh, Saudi Arabia. Participants in the study were parents with children less than 14 years of age who lived in Riyadh, Saudi Arabia, irrespective of whether they were Saudi or non-Saudi. Exclusion criteria included childless families, parents with children diagnosed with epilepsy, and parents not raising their children in Saudi Arabia. The study was conducted from January 2023 to May 2023 in Riyadh.

Using convenience sampling, a self-administered paper questionnaire translated from English into Arabic was distributed among parents. Informed consent and authenticity declaration were embedded at the beginning of the questionnaire. The questionnaire consisted of four sections. Section 1 on sociodemographic characteristics gathered information about parental status, age, nationality, city of residence, number of children, level of education, and job. Section 2 assessed the level of knowledge of FC. Section 3 tested parents’ attitudes toward FCs. Finally, section 4 surveyed parent practices. The demographic section was changed to fit our study population.

Sample size estimation

By assuming that the prevalence of parents with inadequate knowledge, attitudes, and practices toward FCs was 50%, the sample size was calculated as n = (z^2^ pq)/d^2^, where Z is 1.96 (95% CI), p is the prevalence (0.5), q = 1 − p (0.5), and d is the margin of error (0.05). The sample size was calculated to be 384 parents.

Questionnaire criteria

The knowledge regarding FC was assessed using an 11-item questionnaire, with the correct answer identified and coded as 1, while the incorrect answer coded as 0. The overall scores of knowledge were calculated by adding all 11 items. The knowledge score had a possible score ranging from 0 to 11 points. The greater the score, the greater the knowledge about FC. By using 50% and 75% as cutoff points to determine the level of knowledge, parents were categorized as having poor knowledge if the score was below 50%, 50% to 75% were categorized as moderate knowledge levels, and above 75% were categorized as good knowledge levels.

The attitude toward FC was assessed using a 10-item questionnaire, with five-point Likert scale categories ranging from “strongly disagreed,” coded as 1, to “strongly agreed,” coded as 4, and “not applicable,” coded as 0. Negative questions were coded reversely to avoid bias in the score. The total attitude was calculated by adding all 10 items. The greater the score, the greater the attitude toward FC. By applying similar criteria of 50% and 75% as cutoff points, parents were considered to have a negative attitude if the score was below 50%, 50% to 75% were considered neutral attitude levels, and above 75% were considered positive attitude levels.

Statistical analysis

Descriptive statistics were computed and reported as frequencies and proportions (%) for qualitative variables and mean ± standard deviation for quantitative variables. The differences in the scores of the knowledge and attitude regarding the socio-demographic characteristics and experiences toward child FC were performed using the Mann-Whitney U-test. Statistical collinearity was measured using the Shapiro-Wilk test and the Kolmogorov-Smirnov test. The knowledge and attitude scores followed a non-normal distribution. Thus, the non-parametric test was applied. The Spearman correlation coefficient was also used to determine the correlation between the knowledge and attitude scores. Statistical significance was identified at p-values <0.05. Data analyses were performed using SPSS version 26 (IBM Corp. Armonk, NY, USA).

Ethical approval and consent to participate

On March 5, 2023, this study was reviewed and approved according to the International Conference on Harmonisation Good Clinical Practice guidelines by the King Fahad Medical City Institutional Review Board (approval number: FWA00018774).

## Results

A total of 415 parents were enrolled. As shown in Table 1, 350 (84.3%) were mothers. Regarding fathers’ age, 192 (46.3%) were aged between 46 and 60 years, while for the mothers’ age, 222 (53.5%) were aged between 31 and 45 years. Regarding education, approximately 294 (70.8%) of fathers were bachelor’s or higher degree holders, and a similar proportion of 308 (74.2%) was noted for mothers with the same degrees. Most of our respondents were Saudis 410 (98.8%), and 234 (56.4%) were government employees. With respect to monthly income, 172 (41.4%) were earning more than 15,000 SAR per month. In addition, 172 (41.4%) owned a house or villa.

**Table 1:** Socio-demographic characteristics of the parents (n = 415). Values are presented as numbers and percentages (%).

Study data	N (%)
Relationship to child	
Father	65 (15.7%)
Mother	350 (84.3%)
Father’s age	
<20 years	04 (01.0%)
20–30 years	19 (04.6%)
31–45 years	151 (36.4%)
46–60 years	192 (46.3%)
>60 years	49 (11.8%)
Mother’s age	
<20 years	01 (0.20%)
20–30 years	42 (10.1%)
31–45 years	222 (53.5%)
46–60 years	134 (32.3%)
>60 years	16 (03.9%)
Father’s educational level	
Secondary or less	27 (03.6%)
High school	94 (22.7%)
Bachelor’s or higher degree	294 (70.8%)
Mother’s educational level	
Secondary or less	15 (03.6%)
High school	92 (22.2%)
Bachelor’s or higher degree	308 (74.2%)
Nationality	
Saudi	410 (98.8%)
Non-Saudi	05 (01.2%)
Occupational status	
Government employee	234 (56.4%)
Private employee	44 (10.6%)
Retired	85 (20.5%)
Unemployed	52 (12.5%)
Monthly income (SAR)	
<5,000	31 (07.5%)
5,000–10,000	87 (21.0%)
11,000–15,000	125 (30.1%)
>15,000	172 (41.4%)
Accommodation type	
Rented apartment	61 (14.7%)
Own apartment	32 (07.7%)
Rent villa/House	45 (10.8%)
Own villa/House	277 (66.7%)

As shown in Table 2, 137 (33%) of parents reported that their child experienced an episode of FC. Of them, 56 (40.9%) stated that their child experienced FC during toddler age (one to three years old), and 122 (89.1%) parents said that they witnessed it. Most parents (381, 91.8%) indicated having no child sibling with a history of FC, while 349 (84.1%) indicated having no family history of FC. According to parents, the most common causes of FC were fever episodes and the child’s age (298, 71.8%).

**Table 2:** Family experiences of febrile convulsions (n = 415). Values are presented as numbers and percentages (%). ^†^: Variable with multiple response answers.

Variables	N (%)
Has your child experienced an episode of febrile convulsion?	
Yes	137 (33.0%)
No	278 (67.0%)
If the answer is yes, how old was your child at the time of the initial FC episode? (n = 137)	
0–6 months	18 (13.1%)
7–12 months	35 (25.5%)
1–3 years	56 (40.9%)
>3 years	28 (20.4%)
Number of seizure episodes experienced by the child (n = 137)	
One	66 (48.2%)
Two	35 (25.5%)
Three	14 (10.2%)
More than three	22 (16.1%)
Have you ever been present during your child’s seizure? (n = 137)	
Yes	122 (89.1%)
No	15 (10.9%)
Number of the child’s siblings with FC history	
None	381 (91.8%)
One	15 (03.6%)
Two	08 (01.9%)
More than two	11 (02.7%)
Number of family members with FC history	
None	349 (84.1%)
One	35 (08.4%)
Two	09 (02.2%)
More than two	22 (05.3%)
Which of the following are the main causes of FC?^†^	
Inheritance	76 (18.3%)
Abnormal conduction of electric current in the brain	116 (28.0%)
Fever episode and child’s age	298 (71.8%)
Child’s predisposition	84 (20.2%)
Eye spirit	25 (06.0%)

As shown in Table 3, the most commonly taken action during FC was rushing the child to a doctor (100, 73%), followed by lowering the body temperature (73, 53.3%), shaking and rousing the child (29, 21.2%), keeping calm (25, 18.2%) and reading Quran to a patient (25, 18.2%). Regarding an action plan for future FC occurrence, the most common was to rush the child to a doctor (94, 68.6%), followed by lowering the child’s body temperature (78, 56.9%) and placing the child on his/her side (54, 39.4%).

**Table 3:** Practice toward children with febrile convulsions (n = 137). Values are presented as numbers and percentages (%). ^†^: Variable with multiple response answers.

Variables	N (%)
Action taken during child FC^†^	
Rush the child to a doctor	100 (73.0%)
Lower the child’s body temperature	73 (53.3%)
Shake and rouse the convulsion child	29 (21.2%)
Keep calm	25 (18.2%)
Read the Quran to the patient	25 (18.2%)
Observe seizure manifestation and duration	22 (16.1%)
No action	20 (14.6%)
Pry the convulsing child’s clenched teeth apart and put something in his/her mouth	19 (13.9%)
Protect the child on a soft and safe surface	13 (09.5%)
Place the child on his/her side	13 (09.5%)
Stimulate the convulsing child	08 (05.8%)
Restrain the convulsing child	05 (03.6%)
Suck discharge from the child’s nose and mouth	05 (03.6%)
Cardiac massage	02 (01.5%)
Attempt to do mouth-to-mouth resuscitation	02 (01.5%)
Cauterization therapy	01 (0.70%)
Action to be taken if a child has another seizure^†^	
Rush the child to a doctor	94 (68.6%)
Lower the child’s body temperature	78 (56.9%)
Keep calm	54 (39.4%)
Place the child on his/her side	41 (29.9%)
Observe seizure manifestation and duration	37 (27.0%)
Protect the child on a soft and safe surface	36 (26.3%)
Reading the Quran to the patient	22 (16.1%)
Pry the convulsing child’s clenched teeth apart and put something in his/her mouth	18 (13.1%)
Stimulate the convulsing child	12 (08.8%)
Cardiac massage	09 (06.6%)
Attempt to do mouth-to-mouth resuscitation	08 (05.8%)
Suck discharge from the child’s nose and mouth	08 (05.8%)
Restrain the convulsing child	07 (05.1%)
Shake and rouse the convulsion child	06 (04.4%)
No action	02 (01.5%)

In the assessment of FC knowledge (Table 4), most parents showed poor knowledge in nearly all knowledge statements. Parents were unable to identify the correct answer for most statements, specifically about electroencephalography (EEG) or computed tomography (CT), i.e., whether it is important (27, 6.5%), whether putting a protective device into the mouth during convulsion is important (34, 8.2%), and whether a recurrence of FC can cause brain damage (37, 8.9%). Only for the question on “FC is epilepsy” did our respondents reach more than 50%, which indicated adequate knowledge. The overall mean knowledge score was 2.39 (SD = 2.22). Based on our criteria, most of our respondents were classified as having poor knowledge (369, 88.9%), 41 (9.9%) had moderate knowledge, and only five (1.2%) had good knowledge levels. Regarding attitude, the highest ratings were seen in the statements related to “More attention and care are needed for a child with FC” (mean score = 3.17), “It is shameful to have a child with FC” (mean score = 3.02), and “Parents should take their children’s temperature frequently” (mean score = 2.98). The overall mean attitude score was 21.4 (SD = 8.89), with negative, neutral, and positive attitudes constituting 146 (35.2%), 205 (49.4%), and 64 (15.4%) respondents, respectively. Pertaining to concerns about FC, the highest ratings were seen in the statement “Seizure in the night” (mean score = 1.98), followed by “Delayed treatment at the next FC episode” (mean score = 1.81), and “Don’t know how to manage my child during the FC episode” (mean score = 1.78).

**Table 4:** Assessment of the knowledge, attitude, and concerns toward a child with febrile convulsions (n = 415). Values are presented as mean ± SD. Knowledge and attitude levels are presented as numbers and percentages (%). ^†^: Reverse-coded question. Attitude toward FC has a category response ranging from “strongly disagree” (coded with 1) to “strongly agree” (coded with 4); “not applicable” is coded with 0. Concern toward FC has a category response ranging from “not concerned at all” (coded with 1) to “extremely concerned”(coded with 4); “not applicable” is coded with 0.

Knowledge statement	Correct answer, N (%)
1. FC is epilepsy (false)	221 (53.3%)
2. Children with FC receive immunizations on schedule (true)	134 (32.3%)
3. FC is rare after age 5 (true)	111 (26.7%)
4. Anticonvulsant drugs are required for every child with FC (false)	110 (26.5%)
5. Every child with FC will have another FC in the future (false)	98 (23.6%)
6. It is necessary to do mouth-to-mouth resuscitation during convulsion (false)	81 (19.5%)
7. It is necessary to restrain the child during convulsion (false)	76 (18.3%)
8. Risk of subsequent epilepsy in FC is rare (true)	65 (15.7%)
9. Recurrent FC will cause brain damage (false)	37 (08.9%)
10. It is necessary to put a protective device into the mouth to prevent tongue injury during convulsion (false)	34 (08.2%)
11. EEG or CT is necessary for every child with FC (false)	27 (06.5%)
Total knowledge score (mean ± SD)	2.39 ± 2.22
Level of knowledge	
Poor	369 (88.9%)
Moderate	41 (09.9%)
Good	05 (01.2%)
Attitude statement	Mean ± SD
1. More attention and care are needed for a child with FC	3.17 ± 1.33
2. It is shameful to have a child with FC^†^	3.02 ± 1.55
3. Parents should take their children’s temperature frequently	2.98 ± 1.24
4. FC can be outgrown	2.57 ± 1.61
5. FC is due to eye spirit^†^	2.25 ± 1.67
6. Folk medicine is also necessary^†^	2.17 ± 1.72
7. FC will become epilepsy^†^	1.56 ± 1.55
8. An FC attack is a life-threatening event^†^	1.53 ± 1.29
9. FC can cause brain damage^†^	1.11 ± 1.17
10. If necessary, lumbar puncture is acceptable^†^	1.07 ± 1.28
Total attitude score	21.4 ± 8.89
Level of attitude	N (%)
Negative	146 (35.2%)
Neutral	205 (49.4%)
Positive	64 (15.4%)
Concern statement	Mean ± SD
1. Seizure in the night	1.98 ± 1.62
2. Delayed treatment at the next FC episode	1.81 ± 1.65
3. Don’t know how to manage my child during the FC episode	1.78 ± 1.52
4. Apt to get a fever	1.72 ± 1.31
5. Further seizure attacks	1.65 ± 1.59
6. Potential brain damage	1.60 ± 1.56
7. FC attack is life-threatening	1.53 ± 1.55
8. Subsequent epilepsy	1.42 ± 1.50
9. Cannot recognize the seizure attack earlier	1.32 ± 1.47
10. Siblings will have FC too	1.03 ± 1.34

**Figure 1:**
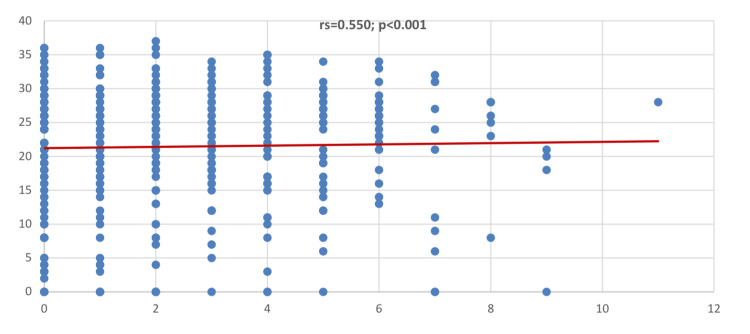
Correlation between knowledge score and attitude score.

As shown in Figure 1, the Spearman correlation coefficient suggested a positive, strong correlation between knowledge score and attitude score (rs = 0.550; p < 0.001).

When measuring the differences in the score of knowledge and attitude in relation to the socio-demographic characteristics and experience of parents toward their child with FC (Table 5), it was observed that a higher knowledge score was more associated with having a child who experienced an episode of FC (Z = 6.123; p < 0.001), having child’s siblings with FC (Z = 3.074; p = 0.002), and having a family history of FC (Z = 1.995; p = 0.046), while a higher attitude score was more associated with being more educated (Z = 2.346; p = 0.019), having better monthly income (Z = 2.036; p = 0.042), having a child with FC (Z = 6.229; p < 0.001) and having a child’s siblings with FC (Z = 3.141; p = 0.002).

**Table 5:** Association between knowledge and attitude according to the socio-demographic characteristics and experiences of parents toward febrile convulsions (n = 415). Values are presented as mean ± standard deviation. ^§^: P-value has been calculated using Mann-Whitney U-test. ^**^: Significant at p < 0.05 level.

Factor	Knowledge score (11), mean ± SD	Z-test; P-value^§^	Attitude score (40), mean ± SD	Z-test; P-value^§^
Relationship to child				
Father	2.25 ± 1.98	0.233; 0.816	20.6 ± 8.41	1.066; 0.287
Mother	2.42 ± 2.26		21.6 ± 8.99	
Father’s age				
≤45 years	2.47 ± 2.34	0.247; 0.805	21.8 ± 8.82	0.767; 0.443
>45 years	2.34 ± 2.13		21.2 ± 8.96	
Mother’s age				
≤45 years	2.43 ± 2.27	0.235; 0.814	21.4 ± 8.82	0.022; 0.983
>45 years	2.33 ± 2.13		21.4 ± 9.06	
Father’s educational level				
High school or less	2.32 ± 2.05	0.021; 0.983	20.0 ± 9.06	2.346; 0.019^**^
Bachelor or higher	2.43 ± 2.29		22.0 ± 8.78	
Mother’s educational level				
High school or less	2.23 ± 2.05	0.645; 0.519	21.3 ± 8.47	0.682; 0.495
Bachelor or higher	2.45 ± 2.27		21.5 ± 9.05	
Occupational status				
Employed	2.38 ± 2.27	0.572; 0.567	21.2 ± 9.09	0.241; 0.810
Unemployed	2.42 ± 2.09		21.8 ± 8.50	
Monthly income (SAR)				
≤10,000	2.29 ± 2.33	1.105; 0.269	19.9 ± 9.39	2.036; 0.042^**^
>10,000	2.44 ± 2.17		22.0 ± 8.64	
Accommodation type				
Rented apartment/villa/house	2.16 ± 2.18	1.444; 0.149	20.3 ± 8.77	1.843; 0.065
Own apartment/villa/house	2.48 ± 2.23		21.8 ± 8.92	
Has your child experienced an episode of FC?				
Yes	3.28 ± 2.25	6.123; <0.001^**^	25.2 ± 6.73	6.229; <0.001^**^
No	1.96 ± 2.07		19.6 ± 9.25	
Having a child’s siblings with FC				
Yes	3.59 ± 2.57	3.074; 0.002^**^	25.6 ± 7.17	3.141; 0.002^**^
No	2.29 ± 2.15		21.1 ± 8.95	
Family history of FC				
Yes	2.92 ± 2.45	1.995; 0.046^**^	22.3 ± 9.30	1.231; 0.218
No	2.29 ± 2.16		21.3 ± 8.82	

## Discussion

This study evaluates parents' knowledge, attitudes, and practices toward a child with FC. Parents’ adequate understanding of FC and their beliefs can positively influence FC episodes at home. Therefore, we conducted this research to shed more light on parents’ views and perspectives on this disease.

FC level of knowledge

The knowledge of parents regarding FC was unsatisfactory. Among 11 items assessing the knowledge, the total mean knowledge score was 2.39 (SD = 2.22), with poor knowledge accounting for 369 (88.9%), only 41 (9.9%) had moderate knowledge, and only five (1.2%) had good knowledge levels. This is almost consistent with the study by AlZweihary et al. [9]. Nearly 70% of the parents living in the Qassim region reported having poor knowledge of FC, while 32.2% had good knowledge levels. Contradicting these reports, a study conducted among Iraqi parents [11] found that almost half (43%) had good knowledge levels, 40% had fair knowledge levels, and only 17% had poor knowledge levels. This has been concurred by the study done in Ghana [12], where 59% of the respondents were knowledgeable about FC. However, most of the applied first-aid resulted in no positive outcomes. The lack of FC knowledge was evident in our study, which requires educational intervention by health authorities.

Significant factors of knowledge

Child experience of FC episode, having a child’s sibling with FC, and having a family history of FC were significant predictors of increased FC knowledge. Eta and Gaelle [13] found a significant association between the knowledge about convulsion in terms of gender and marital status but an insignificant association with age and education. In our study, socio-demographic variables such as parents’ gender, age, education, occupational status, monthly income, and accommodation type were found to have no significant association with knowledge (p > 0.05). This was also observed in a study by Dogahe et al. [3], with knowledge between control and case groups yielding no significant differences in terms of the socio-demographic variables (p > 0.05).

Specific assessment of knowledge

Among the 11 items that measured FC knowledge, the one item that our parents seemed to understand well was “FC is epilepsy,” wherein 221 (53.3%) parents disagreed. The rest of the knowledge items (10 items) were rated below satisfactory levels. Poor ratings were noted for incorrect perceptions about the importance of EEG or CT to children with FC (27, 6.5%), the wrong belief of putting a protective device into a child’s mouth (8.2%), and the wrong perception that FC recurrence would cause brain damage to a child (8.9%). In a study from Iraq [11], 52% of the respondents considered febrile seizures (FS) as correspondent to epilepsy, and 69% believed it was life-threatening. Among Indonesian mothers [14], 61% believed that children’s high fever could lead to seizure events that may cause brain damage (50%) or paralysis (50%).

Knowledge about the causes of FC

Regarding parents’ understanding, fever episodes and the child’s age were the major causes of FC. This mirrored the study by Alfhaid et al. [15], where 53.7% of the respondents were aware that fever recurrence and the child’s age were the most prominent causes of FC. In a study from India [16], some parents wrongly believed that convulsion is just shivering (20.9%), lethargy (20%), excessive cry tantrums (10.9%), fainting spells (8.2%), or evil effect (7.2%). However, in a survey done in Japan [8], more mothers of children with a history of FS reported that high fever caused FS, and antipyretics thwarted the disease from worsening and warmed the child’s body during fever episodes.

FC level of attitude

Over one-third (146, 35.2%) of the respondents were categorized as having a negative attitude, 205 (49.4%) were neutral, and 64 (15.2%) had positive attitude levels (mean score = 21.4; SD = 8.89, out of 40 points). These results were better than the study done in Qassim [9], with 57% reporting negative attitudes and 43% reporting positive attitude levels.

Significant factors of attitude

Increased attitudes were seen more frequently among parents with better education and higher monthly income. Similarly, having a child or child’s siblings with FC greatly increased parents’ attitude levels. Furthermore, there was a strong significant positive correlation between attitude and knowledge scores (p < 0.001), suggesting that the increase in knowledge will also likely increase their attitudes toward FC. In a study by Kausar et al. [17], who measured the knowledge, attitude, and practice after education intervention, a post-test test revealed a significant improvement in mothers’ knowledge, attitude, and practice of FC. The authors emphasized that “educational sessions” are important to reduce parenteral apprehensions and improve parents’ knowledge when managing fever and febrile conditions among their children.

Specific assessment of attitude

Regarding the specific assessment of attitude, respondents in our study had better ratings with some of the attitude determinants, such as “increased attention to be given to a child with FC” (mean score = 3.17 out of 5), “It is shameful to have a child with FC” (mean score = 3.02 out of 5), and taking children’s temperature more frequently (mean score = 2.98 out of 5). In contrast, parents were skeptical regarding the statements “If necessary, lumbar puncture is acceptable” (mean score = 1.07 out of 5), “FC can cause brain damage” (mean score = 1.11 out of 5), and “An FC attack is a life-threatening event” (mean score = 1.53 out of 5). Among Indian parents [18], the most common immediate effects of convulsion were fear of death, insomnia, anorexia, crying, and fear of epilepsy. Incidentally, fear of brain damage, fear of recurrence, and dyspepsia were mentioned alone by children’s fathers. Surprisingly, a relatively high proportion of parents did not know that convulsions can occur due to fever which was consistent with the study done in Ghana [12].

Practice toward a child with FC

The action that needs to be taken during an FC episode is crucial to suffering patients. In our study, nearly three-quarters (100, 73%) of our respondents rushed their child to a doctor, and 73 (53.3%) lowered the child’s body temperature as the most common action taken during FC occurrences. Other actions taken had a lesser rating, most notably on cauterization therapy (1, 0.7%), mouth-to-mouth resuscitation (2, 1.5%), cardiac massage (2, 1.5%), sucking discharge from the child’s nose and mouth (5, 3.6%), restraining the convulsing child (5, 3.6%), and stimulating the convulsing child (8, 5.8%). These results reflected on their planned actions, wherein rushing the child to a doctor (94, 68.6%), lowering the child’s body temperature (78, 56.9%), and keeping calm (54, 39.4%) were the most prominent ones. In Cameroon [13], most parents (99%) stated that they would rush the child to the hospital, 97% agreed to lower the child’s body temperature, and 92% would give paracetamol syrup. However, 92% resorted to a different approach, such as opening a child’s clenched teeth to put something in the mouth, and 67% restrained the convulsing child. Surprisingly, Wuni et al. [12] disclosed that 69% of the parents applied cold water and bathed their children as an FC first aid measure. Further, cultural practices were another method of first-aid for some parents when treating convulsions at home, including smearing ground garlic on children’s bodies during convulsive episodes.

Concerns of FC

Most parents’ concerns toward FC were mainly related to “seizures episode at night,” “delayed treatment at the next FC episode,” and “not knowing how to manage a child during future FC episodes.” In contrast, “siblings will have FC too,” “cannot recognize the seizure attached earlier,” and “subsequent epilepsy” were the least common concerns among parents. In Riyadh [9], potential death during a seizure was the most prominent concern of parents (49.2%), followed by brain damage (44.3%) and epilepsy (42.6%). In India [18], long-term concerns included fear of epilepsy (45.7%) and future recurrence (19.3%) in the affected child; in addition, every subsequent episode of fever was a nightmare for 40% of the parents.

Limitations

This cross-sectional study has several potential limitations that should be noted. First, the data on parents’ knowledge, attitudes, and practices regarding FC in children were self-reported, which is subject to recall bias. Parents may have problems accurately reminiscing their knowledge or attitude, and their reactions may be affected by social desirability, leading to the overreporting of positive attitudes and underreporting of negative attitudes.

Furthermore, this study had a reasonable number of parents, delivering a representative selection of parents in the general population with or without children with FCs in Riyadh, Saudi Arabia, It is essential to mention that the generalizability of the results may be limited. The selection may not entirely illustrate the population, and there could be deviations in knowledge, attitudes, and practices among different subgroups that were not sufficiently apprehended in the study.

Finally, as this was a cross-sectional study, only associations between variables could be considered, and no causal associations could be implied. Longitudinal studies or experimental designs are required to determine causal considerations in knowledge, attitudes, and practices regarding parents with or without children with FCs.

## Conclusions

Despite a modest attitude toward FC, the parents’ knowledge about it appears to be lacking. Parents who had one or more children with FC were seen to have a better perspective toward FC, while parents who were highly educated and had higher earnings were likely to have a better attitude when dealing with a child with FC. Parents had greater concerns about night episodes and delayed treatment. Therefore, proper parental education is necessary to educate parents about the basic facts of FC and the appropriate action to be taken during FC episodes. The parental fear of FC is a major problem affecting their psychological and familial life. Hence, a good understanding of the disease can eradicate their fears and can achieve better management of child FC.
